# Editorial Notes

**Published:** 1882-01

**Authors:** 


### EDITORIAL NOTES.

Malignant scarlatina is prevailing in the eastern portion of our city to quite an alarming extent.

Dr. B. H. Grove has left for Europe, to prosecute his studies in ophthalmology and otology, to which specialty he intends to devote himself in the future.

We learn that our talented young friend Dr. F. Peterson is in Vienna, studiously engaged in the lectures and clinics of that great medical centre, and improving the vast advantages there offered.

The many friends of Dr. Miner will be gratified that his health is so far restored that he has been able to deliver his lectures on Special Surgery, and to attend to the surgical clinics at the Hospital.

We offer no apology for furnishing among our selections the able paper on "Puerperal Hemorrhage," by Prof. Thomas. The subject and the manner in which it is presented by the scholarly writer, call for an attentive study by our readers.

The Report of the Engineers to whom the plans of the Great Trunk Sewer were submitted, affords renewed hope that this work, demanded by the sanitary condition of Buffalo, will not be long delayed. The sewer should be constructed as soon as possible. Human life is more valuable than the millions which the sewer may cost.

The changes in our municipal government, with the advent of the new year, will retire Dr. A. H. Briggs, the Health Physician, from the direction of the sanitary interests of the city. We take pleasure in bearing our testimony to the zeal and energy of Dr. Briggs in the position he has filled for the past two years. Under a very inefficient Health Board, it is a matter of surprise that so much has been done to guard the sanitary interests of the city, for which great credit is due the retiring Health Officer.

The lectures at the Buffalo Medical College are progressing favorably, with a large and excellent class in attendance. In our editorial columns we have noticed the appointment of Dr. Mann, of Hartford, Conn., as Lecturer on Obstetrics. Prof. Doremus has completed his course in chemistry, and left for New York. Dr. Andrews has closed his admirable lectures on Insanity. Dr. Lothrop finished his lectures on Hygiene on the 12th. Prof. Mason is occupying the latter half of the term with daily lectures on Physiology.

